# Calcium Oxide Nanoparticles Have the Role of Alleviating Arsenic Toxicity of Barley

**DOI:** 10.3389/fpls.2022.843795

**Published:** 2022-03-11

**Authors:** Muhammad Mudassir Nazir, Qi Li, Muhammad Noman, Zaid Ulhassan, Shafaqat Ali, Temoor Ahmed, Fanrong Zeng, Guoping Zhang

**Affiliations:** ^1^Department of Agronomy, College of Agriculture and Biotechnology, Zhejiang University, Hangzhou, China; ^2^Institute of Biotechnology, Zhejiang University, Hangzhou, China; ^3^Department of Environmental Sciences and Engineering, Government College University Faisalabad, Faisalabad, Pakistan; ^4^Department of Biological Sciences and Technology, China Medical University, Taichung, Taiwan; ^5^School of Agriculture, Yangtze University, Jinzhou, China

**Keywords:** antioxidants, arsenic, barley, calcium oxide, nanoparticles

## Abstract

Arsenic (As) contamination in agricultural soils has become a great threat to the sustainable development of agriculture and food safety. Although a lot of approaches have been proposed for dealing with soil As contamination, they are not practical in crop production due to high cost, time-taking, or operational complexity. The rapid development of nanotechnology appears to provide a novel solution to soil As contamination. This study investigated the roles of calcium oxide nanoparticles (CaO NPs) in alleviating As toxicity in two barley genotypes (LJZ and Pu-9) differing in As tolerance. The exposure of barley seedlings to As stress showed a significant reduction in plant growth, calcium and chlorophyll content (SPAD value), fluorescence efficiency (*Fv/m)*, and a dramatic increase in the contents of reactive oxygen species (ROS), malondialdehyde (MDA) and As, with LJZ being more affected than Pu-9. The exogenous supply of CaO NPs notably alleviated the toxic effect caused by As in the two barley genotypes. Moreover, the expression of As transporter genes, that is, *HvPHT1;1*, *HvPHT1;3*, *HvPHT1;4* and *HvPHT1;6*, was dramatically enhanced when barley seedlings were exposed to As stress and significantly reduced in the treatment of CaO NPs addition. It may be concluded that the roles of CaO NPs in alleviating As toxicity could be attributed to its enhancement of Ca uptake, ROS scavenging ability, and reduction of As uptake and transportation from roots to shoots.

## Introduction

Arsenic (As) is a ubiquitous heavy metalloid that imposes severe toxic effects on living organisms (Zhang et al., 2021). As exists in the soil in the forms of arsenate (As^V^) and arsenite (As^III^; Kanwar and Bhardwaj, 2015). As^V^, being a chemical analog of phosphorus (P), enters root cells *via* phosphate transporters (PHTs) located in the root epidermis (Zvobgo et al., 2019), while As^III^ utilizes aquaporin channels for its transportation to aerial plant parts (Clemens and Ma, 2016). Plants may accumulate a surplus amount of As from soil, causing phytotoxic effects, such as inhibition of plant growth, photosynthesis and biomass production, limitation of nutrient supply, cellular damage, and disturbance of cellular redox homeostasis (AbdElgawad et al., 2021b). As the arsenic level in soil and water systems has been increasing because of anthropogenic activities (Abbas et al., 2018), effective strategies for fighting against its toxicity should be performed to ensure sustainable development of agriculture production and food safety. Although there are a lot of approaches proposed for dealing with soil As contamination, they are impractical due to high capital investment, time-taking, or operational complexity (Alka et al., 2021).

Nanotechnology has emerged as a promising technique for achieving long-term agricultural sustainability due to its several advantages for the agro-ecosystem, including increasing nutrient utilization efficiency, alleviating the impacts of climate change, and remediating heavy metals/metalloids (Manzoor et al., 2021). Recently, calcium oxide nanoparticles (CaO NPs) have gained great attention worldwide because of their promising agricultural applications. The positive effects of calcium NPs on the growth of Bengal gram (*Cicer arietinum* L.; Gandhi, 2021), lettuce (*Lactuca sativa*), zucchini (*Cucurbita pepo*; Meier et al., 2020), and rice (*Oryza sativa*; Syu et al., 2020) have been reported. In a recent study, Ahmed et al. (2021a) revealed that the application of magnesium oxide NPs significantly reduced As uptake in roots and shoots by improving rice plant growth and cellular antioxidant content. Similarly, Ma et al. (2020) reported that zinc oxide NPs reduced As concentration in rice roots (39.5%) and shoots (60.2%), and the reduction was primarily due to the lowered inorganic As (III) and organic As species. Although various NPs have been used as nano-fertilizers to improve plant growth and development by reducing As toxicity, such as zinc oxide NPs (Yan et al., 2021) in rice, titanium oxide NPs in mung bean (Katiyar et al., 2020), and zinc oxide NPs in soybean (Ahmad et al., 2020), the roles of CaO NPs in alleviating As-induced crop toxicity are still elusive.

Barley (*Hordeum vulgare* L.) is one of the most important crops globally, with multiple uses (Zhang and Li, 2010; Han et al., 2020). Moreover, it is also an ideal crop for producing healthy food as barley grains contain high content of β-glucan, flavonoid, and bioactive compounds. Meanwhile, barley is considered as a classical model plant for physiological and genetic studies on cereal plants (Gürel et al., 2016). Barley is more sensitive to As toxicity in comparison with other cereal crops (Baghaie and Jabari, 2019). Therefore, it is imperative to reduce As uptake and accumulation in barley plants exposed to As-contaminated soils.

The objectives of the current study were to determine the beneficial effects of CaO NPs on growth and physiological traits of two barley genotypes differing in As tolerance under As stress. In addition, the roles of CaO NPs in reducing As bioavailability and acro-petal translocation through its regulation of *HvPHTs* was also examined, so as to determine if CaO NPs can be used as a nano-fertilizer to alleviate As toxicity and accumulation in barley.

## Materials and Methods

### Characterization of CaO NPs

In the present study, CaO NPs (purity, 99.9%; size, 30–50 nm) was purchased from Chaowei Nanotechnology Co. (Shanghai, China). The surface morphology and size of CaO NPs were determined by scanning electron microscopy (SEM; SU-8010, Tokyo, Japan) and transmission electron microscopy (TEM; JEM-1230, Akishima, Japan). The samples were prepared on a carbon-coated Cu grid and aluminum stub for both SEM and TEM analysis, according to Ahmed et al. (2021a). X-ray diffraction (XRD) analysis was performed to characterize the crystalline structure of CaO NPs using an X-ray diffractometer (Bruker, Germany). The particle size of CaO NPs was calculated using Debye Scherrer’s equation (*d* = Kλ/β cosθ). Fourier transform infrared spectroscopy (FTIR, Bruker, Germany) was performed to analyze the functional groups of CaO NPs with a spectral range of 500–4,000 cm^−1^. The metallic fractions and elemental compositions of CaO NPs were characterized by energy dispersive spectroscopy (EDS) at 20 KeV.

### Plant Materials and Growth Conditions

Two barley genotypes, that is, Longjiangzao (LJZ; As high accumulator/sensitive) and Pu-9 (As low accumulator/tolerant), were used according to our previous screening experiment (data not shown). The seeds were surface-sterilized by soaking in 5% NaClO solution for 30 min and washed five times with sterile deionized water. For germination, the sterilized seeds were placed in petri dishes on wet filter paper for 24 h and then transferred to a 5 L box containing basic solution medium [BSM: 1 mM KNO_3_ + 0.05 mM Ca (NO_3_)_2_] and covered with a plastic plate with 100 evenly spaced holes. After 3 days, healthy and uniform seedlings were selected and transplanted into new 5 L pots covered with plastic plates with 7 evenly spaced holes (2 plants/hole). Each pot was filled with barley nutrient solution containing (mg l^−1^): 48.2 (NH_4_)_2_PO_4_, 154.8 MgSO_4_.7H_2_O, 24.8 NaH_2_PO_4_, 101.1 KNO_3_, 118.08 Ca (NO_3_)_2_.4H_2_O, 7.6 EDTA Fe Na, 0.9 MnCl_2_.4H_2_O, 2.9 H_3_BO_3_, 0.015 Na_2_MoO_4_.2H_2_O, 0.11 ZnSO_4_.7H_2_O and 0.04 CuSO_4_.5H_2_O. pH was adjusted to 5.6 ± 1 using NaOH or HCl as required. Barley seedlings were exposed to different As concentrations (0, 25, 50, 100, 150 μm). The selection of As concentration for further experimentation was based on this primary experiment, which showed that 50 μM As caused the significant damage to both barley genotypes. The nutrient solution in each pot was continuously aerated with air pumps and renewed twice a week. At the 7th day after seedlings transplanting (14 plants/pot), As (Na_2_HAsO_4_.7H_2_O) and CaO NPs were applied to the corresponding pots to form the 4 treatments: (1) control (CK); (2) 50 μM As; (3) 25 mg/l CaO NPs; (4) 50 μM As +25 mg/l CaO NPs. CaO NPs were sonicated for 1 h before use, which was beneficial for dispersing CaO NPs in hydroponic solution. The experiment was arranged in a complete randomized design with four replicates for each treatment.

At the 7th day after treatment, plants were sampled from each treatment, and plant height, root lengths, and fresh weights were measured immediately. Then metal ions possibly attached on the root surface were removed by soaking roots into the solution containing 20 mm EDTA (Ethylene Diamine Tetra Acetic acid) for 30 min, washing with distilled water, and dried with filter papers. Then these plants were divided into shoots and roots, and some of them were frozen in liquid N_2_ and stored in a −80°C refrigerator for further use. Some of the plant tissues were dried in an oven (60°C) to constant weight for biomass determination.

### Chlorophyll Content and Fluorescence

The chlorophyll content of barley leaves was measured by a portable device (SPAD-502+, Tokyo, Japan). Chlorophyll fluorescence (*Fv/m*) was measured using a portable fluorimeter (OS-30p+, Hudson, United States). The plants were placed in dark for 30 min before measurement, and quantum efficiency of photosystem II (*F_v/m_* = (*F_m_*–*F_o_*)/*F_m_*) was observed at solid-state light (660 nm) source with the intensity of 1,100 μmolm^−2^·s^−1^ as described by Cai et al. (2020).

### As and Ca Content in Plant Tissues

Dry root and shoot samples (0.5 g) were digested in HNO_3_ solution at 120°C for 1 h, followed by 140°C for 2 h in a dry thermos unit (DTU-2CN; Tokyo, Japan). Then the digested solution was diluted with double-distilled water to reach 10 ml as the final volume. As and Ca contents were measured using an inductively coupled plasma mass spectrometer (ICP-MS, iCAP RQ, Thermo scientific, United States). In addition, As accumulation and translocation factors were calculated according to Zhang et al. (2020).

### Reactive Oxygen Species, Malondialdehyde, and Histochemical Analysis

This study measured reactive oxygen species in plant tissues, including hydrogen peroxide (H_2_O_2_) and superoxide-free radical ($O_{2}^{•-}$). Hydrogen peroxide content was measured as described by Velikova et al. (2000). Briefly, barley shoot and root samples (0.2 g) were homogenized in trichloroacetic acid (0.1% TCA) and centrifuged at 13,000 rpm for 15 min. Then supernatant (50 μl), 1 M KI (100 μl), and 10 mM potassium phosphate buffer (50 μl-pH 7.0) were mixed and placed at a microplate reader (Synergy H1 Bio-Tec). H_2_O_2_ content was recorded at 390 nm wavelength.

Superoxide-free radical content was measured according to Schneider and Schlegel (1981). Briefly, 0.2 g of tissue samples was homogenized with 65 mm potassium phosphate buffer (pH 7.8) and centrifuged at 5,000 rpm for 10 min at 4°C. Afterward, 1 ml supernatant was mixed with 0.1 ml of 10 mm hydroxylamine hydrochloride and 0.9 ml of 65 mM potassium phosphate buffer (pH 7.8) and incubated at 25°C for 20 min. Then 1 ml of 17 mM 4-aminobenzenesulphonic acid (C_6_H_7_NO_3_S) and 1 ml of 7 mm a-naphthylamine (C_10_H_7_NH_2_) were added, gently shaked, and incubated again for an additional 20 min at 25°C. Finally, 3 ml trichloromethane (CHCL_3_) was added to the samples and the absorbance was recorded at 530 nm.

Malondialdehyde (MDA) content was determined according to Morales and Munné-Bosch (2019). Briefly, 100 mg of plant tissues were homogenized with chilled potassium phosphate buffer (65 mm; pH 7.8) and centrifuged at 12,000 rpm at 4°C for 15 min. The reaction solution of 5% trichloroacetic acid (TCA) and thiobarbituric acid (TBA) was added to the resultant supernatant and incubated at 95°C for 25 min. Afterward, samples were placed on ice to stop the reaction and centrifuged at 4,800 rpm for 10 min, and specific and non-specific absorbance were recorded at 600 and 532 nm, respectively.

Accumulation of H_2_O_2_ and $O_{2}^{•-}$ in plant tissues was identified by leaf or root staining with 3, 3-diaminobenzidine (DAB) and nitro blue tetrazolium (NBT), respectively, according to Romero-Puertas et al. (2004). The blotted leaves and roots were photographed using a digital microscope (Leica MZ-g5, Germany).

### Antioxidant Enzymatic Activities

The root and shoot samples were homogenized in sodium phosphate buffer (pH 7.8), centrifuged at 13,000 rpm for 20 min at 4°C. Then superoxide dismutase (SOD) activity was measured spectrophotometrically as described by Zhang et al. (2008), at 560 nm by assessing the ability of each unit to inhibit 50% photochemical reduction of nitro blue tetrazolium chloride (NBT). Peroxidase (POD) activity was determined according to Zhou and Leul (1999), at 470 nm and the changes related to guaiacol were normalized with the activity constant (*ε* = 26.6 mm cm^−1^). and catalase (CAT) activity was determined according to Aebi (1984).

### RNA Extraction and Quantitative Real-Time PCR Assay

Total RNA was extracted from barley tissues (root and shoot) using the Steady Pure Universal RNA Extraction Kit (Accurate Biotechnology, Hunan, China) according to the manufacturer’s instructions. The RNA quantity and quality were determined using a Nanodrop and 2% agarose gels, respectively. To synthesize the cDNA, an Evo M-MLV RT Kit (AG 11706, Accurate Biotechnology, Hunan, China) with gDNA Clean for qPCR was used following the manufacturer’s instructions. The amplification reaction for qRT-PCR was performed by using 1 μl of cDNA, 0.8 μl of forward and reverse primers, 10 μl SYBR Green Pro Taq HS premix (AG 11701, Accurate Biotechnology, Hunan, China), and 7.4 μl RNA-free water to reach the final volume up to of 20 μl. All cDNA samples were performed in triplicate by qRT-PCR in a Light Cycler 480 II (Roche, Diagnostics system, Basel, Switzerland). The PCR profile was as follows: initial denaturation 95°C for 30 s, followed by 40 cycles and denaturation at 95°C for 5 s, annealing at 60°C for 30 s. Specific primers for PHT genes were designed using Primer-BLAST (Supplementary Table S1).^1^ Barley ACTIN gene was used as a reference gene and subtracted from the threshold cycle (Ct) values for each sample. Quantitative relative gene expression levels were determined by following the 2^−ΔΔCT^ method according to Schmittgen and Livak (2008).

### Statistics Analysis

Data analysis was performed with the Statistics 8.1 software package. The significance between treatments was tested using two-way ANOVA and compared by LSD test at *p* < 0.05.

## Results

### Characterization of CaO NPs

**Figure 1:**
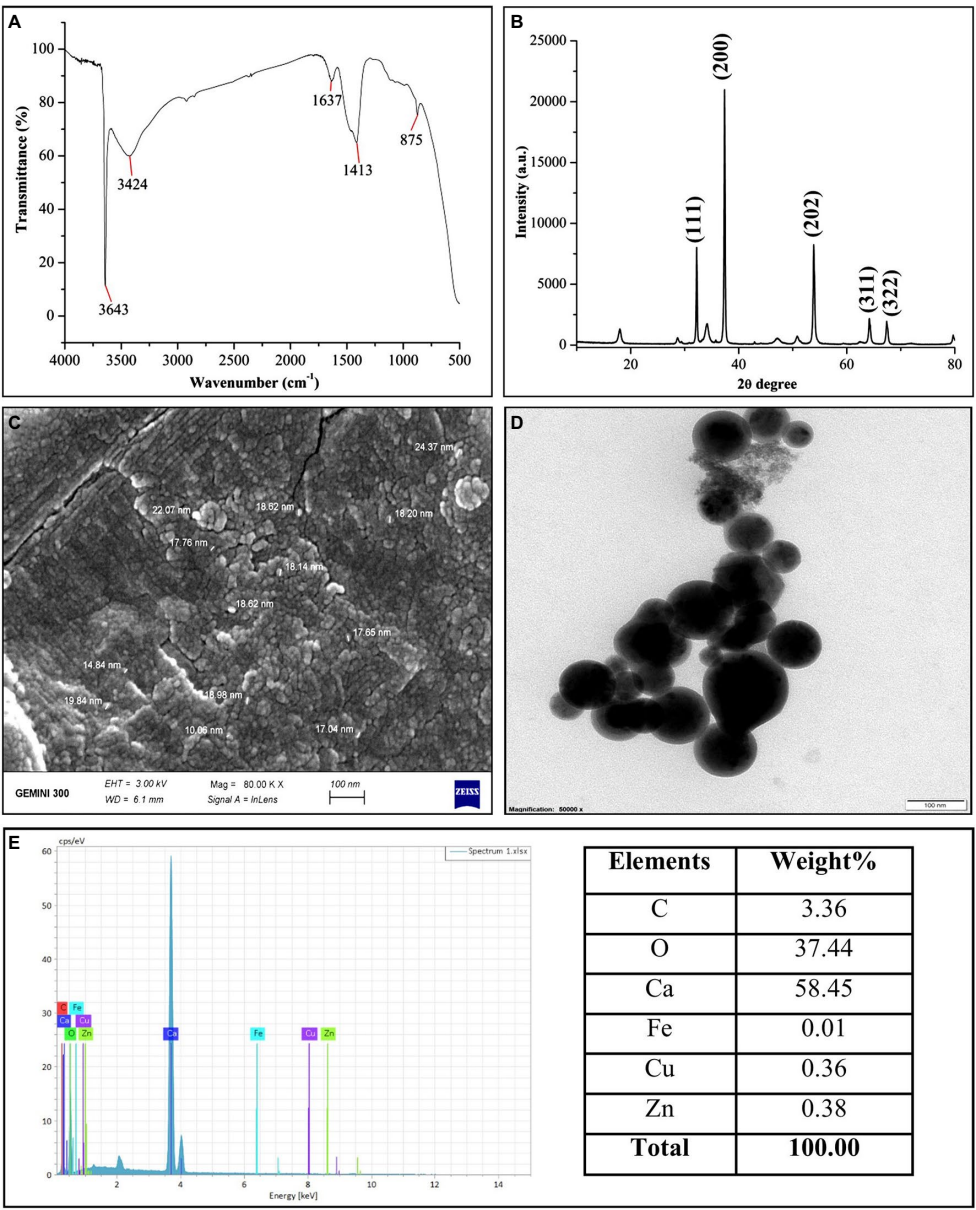
Characterization of CaO NPs. **(A)** FTIR; **(B)** XRD; **(C)** SEM (scale bar = 100 nm); **(D)** TEM (scale bar = 100 nm); **(D)** EDS.

The presence of capping agents on the CaO NPs surface was confirmed through FTIR analysis (Figure 1A). Multiple spectral bands (3,643, 3,424, 1,637, 1,413, 875 cm^−1^) in the FTIR spectrum demonstrated the presence of diverse functional groups as capping agents around the surface of CaO NPs. The presence of strong spectral bands at 3,643 and 3,424 cm^−1^ confirmed the O-H stretching of the alcohol group. The medium peaks at 1,637, 1,413, and 875 cm^−1^ corresponded to C=C stretching, O-H bending, and C-H bending, respectively. The XRD spectrum of CaO NPs revealed the crystalline structure of CaO NPs. The various diffraction peaks of CaO NPs were observed at 32.2, 37.3, 53.8, 64.2, and 67.4 ^o^, which corresponds to 111, 200, 202, 311, and 322 diffraction planes of CaO NPs (Figure 1B), respectively. The SEM and TEM analysis showed that CaO NPs have spherical shapes and variable sizes ranging from 10 to 24 nm (Figure 1). However, CaO NPs were not well dispersed and appeared in nanopowder as aggregated form. Furthermore, EDS spectra confirmed the existence of carbon (3.36%), oxygen (37.54%), calcium (58.54%), iron (0.01%), copper (0.36%) and zinc (0.38%) in CaO NPs (Figure 1E).

### Plant Growth Traits

**Figure 2:**
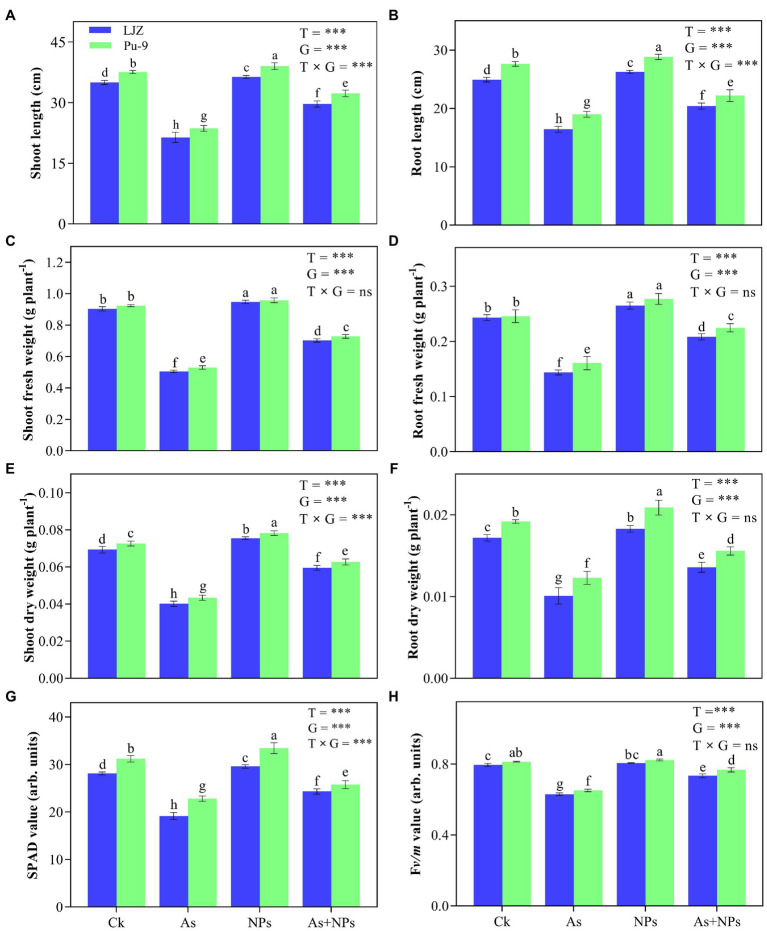
Effects of different treatments on plant growth and chlorophyll contents. **(A,B)** shoot and root length; **(C,D)** fresh weight of shoots and roots; **(E,F)** dry weight of shoots and roots. **(G)**, SPAD value; **(H)**, maximum quantum efficiency (Fv/m) of photosystem II. Vertical bars represent the mean ± SD of four independent replicates. Different letters above error bars indicate the significant difference between treatments and genotypes at *p* ≤ 0.05. T, treatment; G, genotype; T × G, the interaction between treatment and genotype.

As treatment significantly reduced plant growth of both barley genotypes, with LJZ being more affected than Pu-9. As-treated plants showed a significant reduction in shoot length (33.4 and 28.2%), root length (27.9 and 23.4%), shoot fresh weight (36.3 and 30.8%), root fresh weight (25.6 and 20.6%), shoot dry weight (15.7 and 12.2%), root dry weight (11.2 and 9.8%) of LJZ and Pu-9, respectively as compared to control plants (Figure 2). The application of CaO NPs displayed a marked improvement in all growth parameters of the two genotypes, with Pu-9 showing greater improvement than LJZ compared to non-treated plants (Figure 2). Compared with As treatment, CaO NPs + As treatment showed much less inhibition in all plant growth traits.

### Chlorophyll Content and Fluorescence

The toxic effects of As and its alleviation by CaO NPs on chlorophyll content and fluorescence of the two barley genotypes are presented (Figures 2G,H). The significant decline of both SPAD value and maximum quantum efficiency (*Fv/m*) was observed in the two barley genotypes, with LJZ being more affected than Pu-9 under As stress as compared to control. Compared to As treatment, the treatment of CaO NPs + As had significantly greater SPAD and *Fv/m* values for both barley genotypes.

### Reactive Oxygen Species Contents and Antioxidant Enzyme Activities

**Figure 3:**
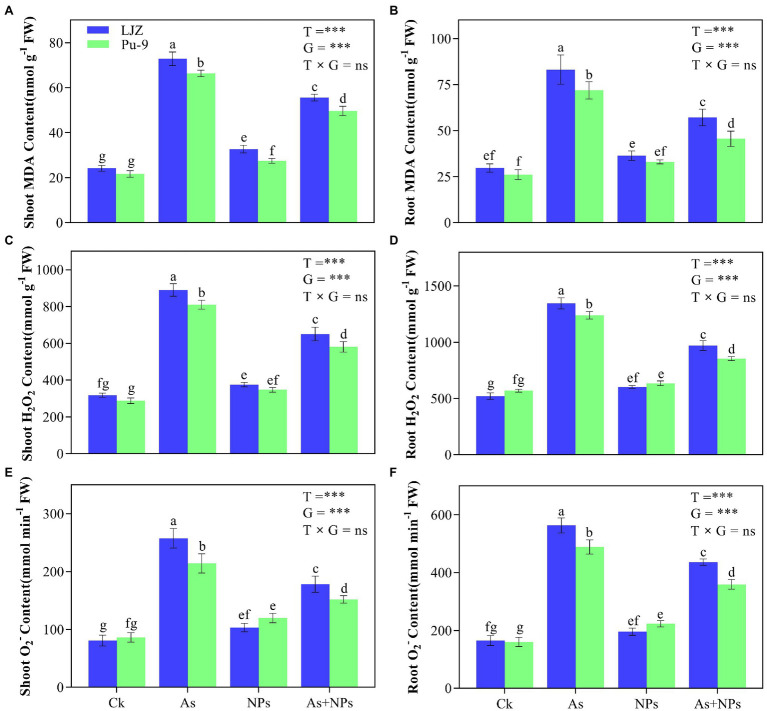
Effects of the different treatments on MDA and ROS (H_2_O_2_ and $O_{2}^{•-}$) contents. **(A,B)** MDA content in shoots and roots; **(C,D)** H_2_O_2_ content in shoots and root; **(E,F)** $O_{2}^{•-}$ content in shoots and roots. Vertical bars represent the mean ± SD of four replicates. Different letters above error bars indicate the significant difference between treatments and genotypes at p ≤ 0.05. T, treatment; G, genotype; T × G, the interaction between treatment and genotype.

**Figure 4:**
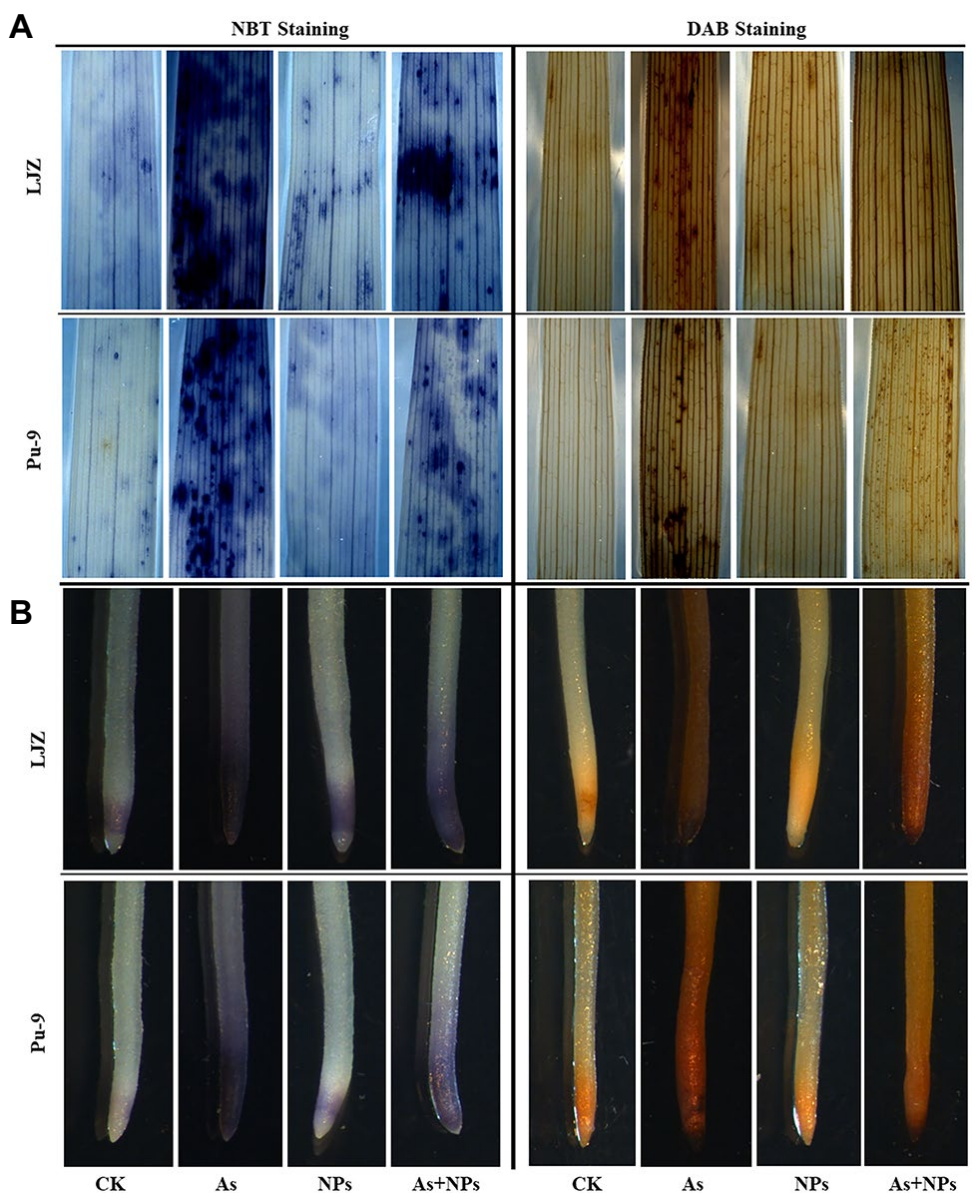
*In situ* histochemical observation of $O_{2}^{•-}$ through NBT and H_2_O_2_ by DAB staining. **(A)** shoot; **(B)** root.

As treatment significantly increased MDA, H_2_O_2_, and $O_{2}^{•-}$ contents in the shoots and roots of the two barley genotypes compared with the control, with LJZ being more affected than Pu-9 (Figure 3). The addition of CaO NPs notably alleviated the oxidative stress in barley tissues caused by As stress, reflected by reduced MDA and ROS contents (Figure 3). In addition, histochemical staining confirmed the results that As stress caused more accumulation of ROS in shoots and roots of both LJZ and Pu-9 and the addition of CaO NPs could alleviate the oxidative stress (Figure 4). Moreover, LJZ had more and darker spots in both shoots and roots than Pu-9 after staining with NBT and DAB, respectively, indicating LJZ accumulated more H_2_O_2_ and $O_{2}^{•-}$ than Pu-9.

**Figure 5:**
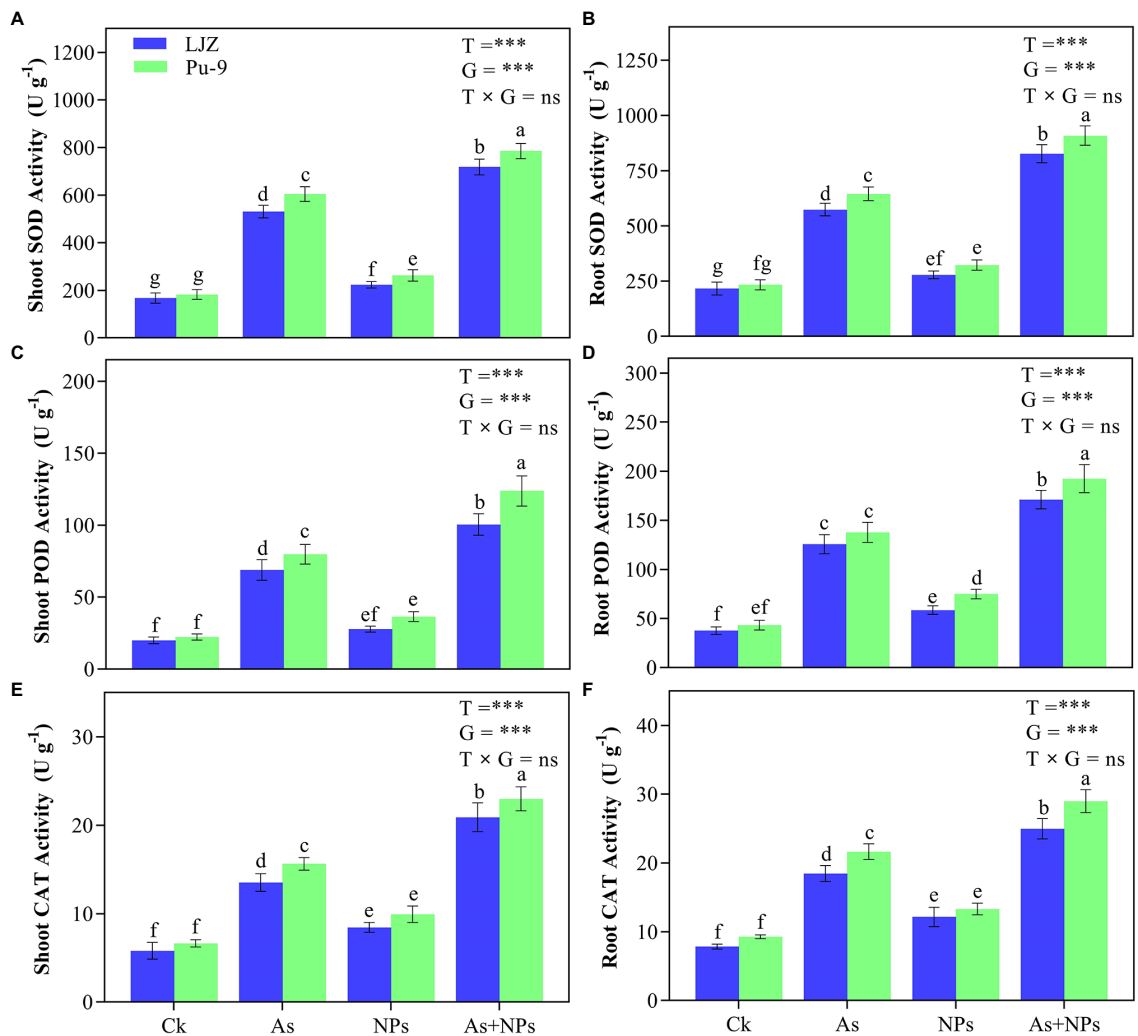
Effects of the different treatments on antioxidant enzyme activities. **(A,B)** superoxide dismutase (SOD) activity in shoots and roots; **(C,D)** peroxidase (POD) activity in shoots and roots; **(E,F)** catalase (CAT) activity in shoots and roots. Vertical bars represent mean ± SD of four replicates. Different letters above error bars indicate the significant difference between treatments and genotypes at *p* ≤ 0.05. T, treatment; G, genotype; T × G, the interaction between treatment and genotype.

For antioxidant enzyme activity, As treatment caused the significant increase of SOD, POD, and CAT in the leaves and roots of both barley genotypes compared with control, with Pu-9 showing more increase than LJZ (Figure 5). The treatment of As + CaO NPs showed significantly higher SOD, POD, and CAT activities in the leaves and roots of Pu-9 and LJZ, compared with As treatment alone.

### Ca, As Content and Translocation

**Figure 6:**
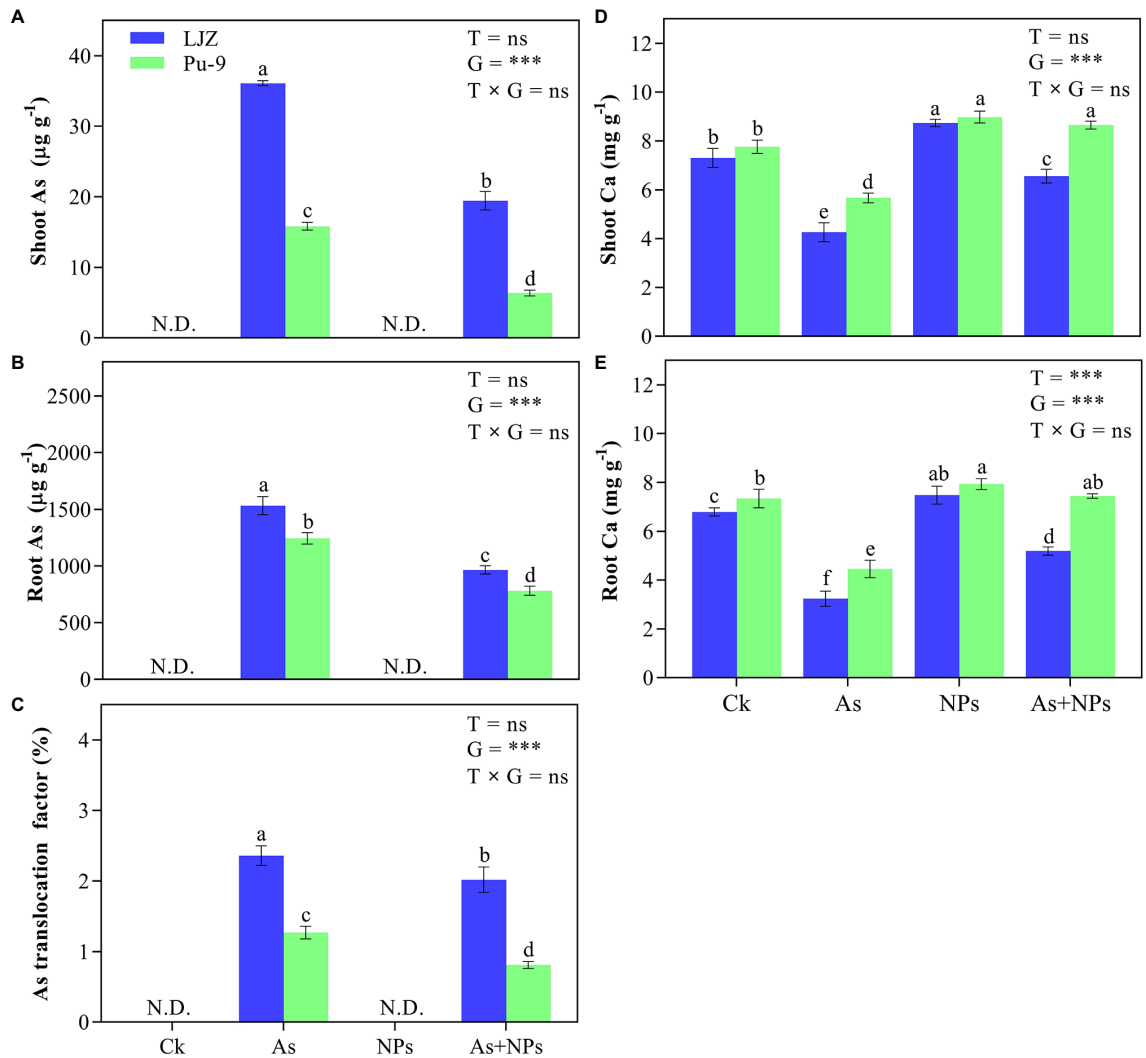
Effects of the different treatments on Ca, As contents, and translocation factor in barley tissues. **(A, B)** As content in shoots and roots; **(C)** translocation factor; **(D, E)** Ca content in shoots nd roots. Different letters above error bars indicate the significant difference between treatments and genotypes at *p* ≤ 0.05. T, treatment; G, genotype; T × G, the interaction between treatment and genotype.

Without As addition in the culture solution (both control and CaO NPs treatment), As content was not detected in both root and shoots of the two genotypes (Figures 6A,B). There was a significant difference in As content between shoots and roots and the two barley genotypes in the As treatment alone. The As + CaO NPs treatment significantly reduced As content in shoots and roots compared with the As treatment alone, with Pu-9 showing more reduction than LJZ. Moreover, As treatment reduced calcium content in plant tissues of both genotypes, which are significantly increased by CaO NPs addition (Figures 6D,E). For As translocation factor (TF) from root to shoots, in As treatment, LJZ (40.8%) was larger than Pu-9 (31.1%). However, CaO NPs + As treatment caused a significant reduction of As TF in both genotypes, although LJZ had a significantly larger TF than Pu-9 (Figure 6C).

### Relative Expression of As Transporter Genes

**Figure 7:**
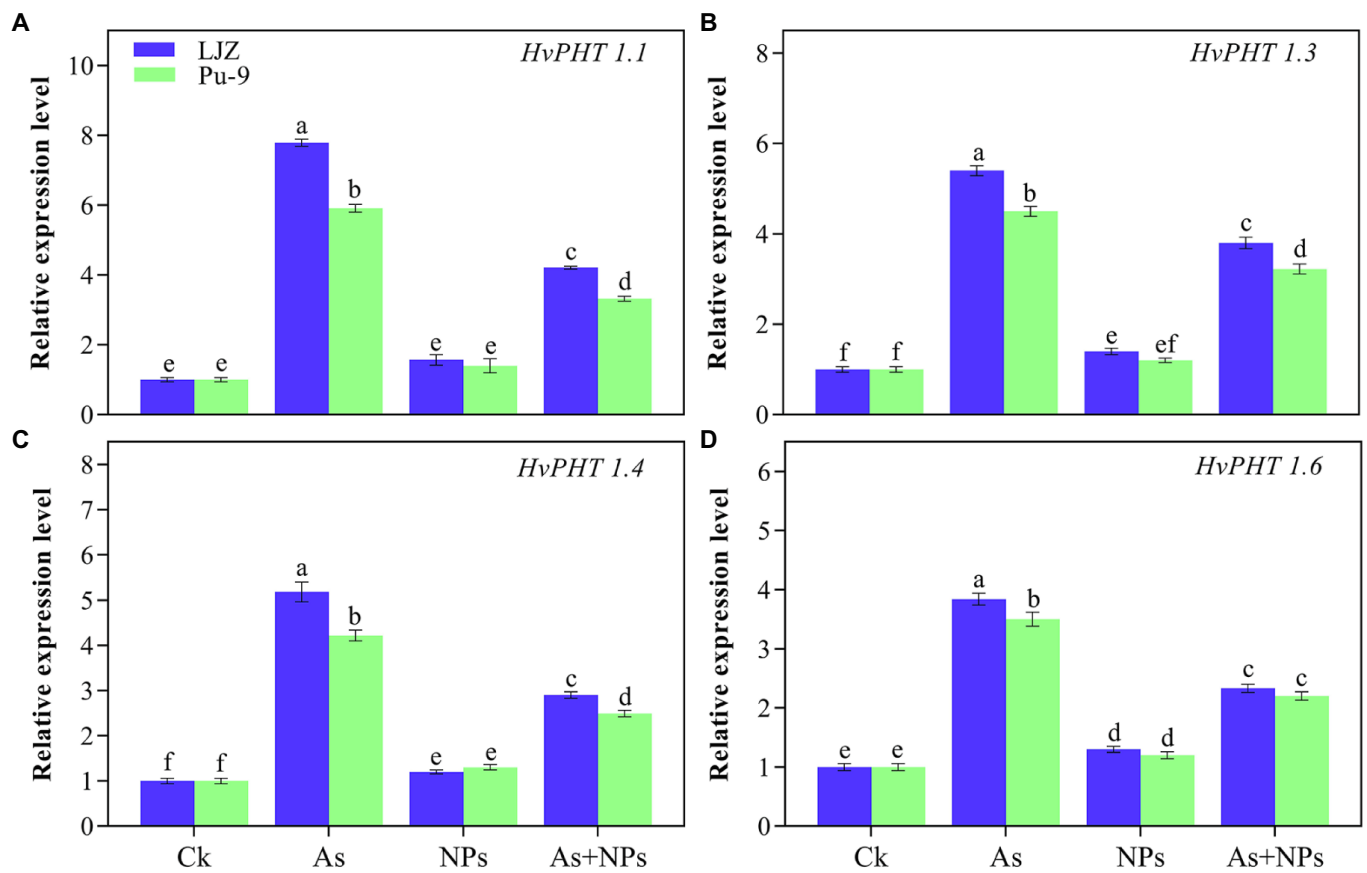
Relative expression of barley phosphate transporter genes **(A)** *HvPHT1.1*, **(B)** *HvPHT1.3*, **(C)** *HvPHT1.4*, and **(D)** *HvPHT 1.6* in roots under As stress. Different letters above error bars indicate the significant difference between treatments and genotypes at *p* ≤ 0.05.

The relative expression of As transporter genes involved in As uptake are presented in Figure 7. In comparison with control, As treatment significantly upregulated the expressions of *HvPHT1;1, HvPHT1;3, HvPHT1;4 and HvPHT1;6* in the two barley genotypes; however, the greater expression was observed in LJZ as compared with Pu-9. The addition of CaO NPs in the As-containing solution caused the significant reduction in the expression of *HvPHT1;1*, *HvPHT1;3*, *HvPHT1;4* and *HvPHT1;6* in comparison with As treatment alone, although the expressions of these four transporter genes were still significantly higher than those in the control plants.

## Discussion

### CaO NPs Alleviate Plant Growth Inhibition Caused by As Stress

As treatment caused a significant reduction in shoot and root lengths, and biomass, with LJZ being more affected than Pu-9, confirming the distinct difference in As stress tolerance between the two barley genotypes. The toxic effect of As stress on plant growth has been intensively studied (Anawar et al., 2018; Singh et al., 2020). AbdElgawad et al. (2021a) reported that barley plants exposed to As stress showed the obvious decline in photosynthesis, which was closely associated with As accumulation in plant tissues and oxidative stress. In recent years, many studies demonstrated that applications of NPs could efficiently reduce As accumulation in plants (Ahmed et al., 2021b,c). In this study, we found CaO NPs significantly improved the growth of barley seedlings exposed to As stress. The alleviation of As toxicity on plant growth by CaO NPs could be attributed to reduce As accumulation in plant tissues and increased photosynthesis, reflected by increased chlorophyll content and fluorescence efficiency.

### CaO NPs Modulate Photosynthetic Efficacy Under Induced As Stress

Photosynthesis is an important indicator of plant adaptation to severe environmental conditions (Hussain et al., 2021). The current results showed that As stress notably reduced chlorophyll content (SPAD value) and photosynthetic efficiency (*Fv/m*) in both barley genotypes (Figure 2). Bidi et al. (2021) reported negative impacts of As on the working efficacy of photosynthetic pigments in rice plants. According to Patel et al. (2018), an increase in chlorophyllase activity and reduction in levels of chlorophyll synthesizing enzymes caused reduced chlorophyll content in As-exposed plants, while the low activity of the photosynthetic apparatus resulted in less sugar production, finally reducing plant biomass (Yan et al., 2021). Obviously, the reduced SPAD and *Fv/Fm* values in the two barley genotypes exposed to As treatment could be attributed to As stress, reflected by higher As contents in the As-treated plants. In this study, exogenous CaO NPs application alleviated the negative impact of photosynthetic efficiency caused by As stress in both barley genotypes (Figure 2). Similarly, the application of ZnO NPs was found to improve the photosynthetic efficiency of wheat plants under cadmium (Cd) stress (Ali et al., 2019; Rizwan et al., 2019) and rice seedlings under As stress (Yan et al., 2021). Moreover, Gandhi et al. (2021) reported that CaO NPs could improve the photosynthesis activity in chickpea by reducing the damage of chloroplast thylakoids and maintaining cellular homeostasis under abiotic stress.

### CaO NPs Reduce ROS Accumulation and Increase Antioxidative Enzyme Activity

Another reason for alleviating As toxicity on barley seedlings by CaO NPs is related to its ability to reduce oxidative stress by improving antioxidative capacity. As treatment significantly induced the extra generation of ROS, which will cause oxidative stress and membrane peroxidation, as reflected by increased MDA content (Zvobgo et al., 2019; Ahmed et al., 2021a). Indeed, we found more MDA, H_2_O_2_, and $O_{2}^{•-}$ contents in the As-treated plants than control and As-tolerant Pu-9 than As-sensitive LJZ (Figures 3, 4). Normally, plants will strengthen their cellular antioxidant defense capacity for responding to oxidative stress, characterized by increased antioxidant enzyme activity. In the present study, As treatment increased SOD, POD, and CAT activities in barley seedlings, accompanied by the increase of MDA, H_2_O_2_, and $O_{2}^{•-}$ contents. Obviously, the increase of these antioxidant enzyme activities is a self-defense response to oxidative stress and is not enough to scavenge the generated ROS. The exogenous application of CaO NPs enhanced SOD, POD, and CAT activities in plant tissues of both barley genotypes (LJZ and Pu-9), accompanied by a decrease in MDA, H_2_O_2_, and $O_{2}^{•-}$ contents in plant tissues, indicating the alleviation of oxidative stress (Figure 5). It was reported that iron oxide NPs increased the activities of antioxidant enzymes (SOD and POD), resulting in a lower accumulation of MDA and H_2_O_2_ in Cd-exposed wheat tissues (Adrees et al., 2020).

Similarly, Ahmad et al. (2020) revealed that the application of zinc oxide NPs significantly improved antioxidative defense capacity and reduced As uptake in rice plants. Moreover, Wu et al. (2020) observed that seed priming with zinc oxide NPs could increase plant biomass, SOD, and CAT activities and reduce As uptake in rice seedlings. It may be suggested that these nanoparticles have the function of enhancing antioxidative stress in plants. However, the underlying mechanism is still elusive.

### CaO NPs Reduce As Accumulation and Increase Ca^2+^ Content in Plant Tissues

In general, plant roots have a higher tolerance to heavy metals than shoots. Hence, less translocation of toxic metals accumulated in roots to shoots is a trait associated with higher tolerance (Cui et al., 2018; Tian et al., 2019; Basit et al., 2022). In this study, we quantified As translocation of roots to shoots in the two barley genotypes and the effect of CaO NPs on As translocation (Figure 6C). As expected, Pu-9 had a significantly smaller As TF than LJZ. Meanwhile, the addition of CaO NPs in the As-containing solution caused a significant reduction of As TF in the two barley genotypes. Hence, it is indicated that alleviation of As toxicity by CaO NPs is also attributed to its role in reducing As uptake and translocation from roots to shoots. A possible explanation is that CaO NPs might immobilize metal ions in the rhizosphere and protect roots by forming coats around the root surface, thus restricting As translocation from roots to shoots.

Furthermore, the possible roles of Ca^2+^ in the effect of CaO NPs in alleviating As toxicity of barley should be noted. Ca^2+^ ions play a vital role in improving plant abiotic stress tolerance by reducing heavy metal ions uptake and modulating the metal transporter genes (Bose et al., 2011). In the present study, we found that the addition of CaO NPs in the culture solution increased Ca^2+^ content in barley tissues, which should be beneficial for the alleviation of As toxicity (Figure 5). Siddiqui et al. (2020) reported the negative correlation between Ca^2+^ content and As metal uptake in *Vicia faba* plants, which was attributed to competing for inhibition of Ca^2+^ ions on As uptake. However, how much role Ca^2+^ ions play in alleviating As toxicity as a part of CaO NPs remains to be clarified.

### CaO NPs Modulate Expression Levels of *HvPHTs* and Reduce Intraplant As Transport

It was reported that plant As toxicity was also associated with the expression levels of *HvPHT* genes, with sensitive genotypes showing higher expression than tolerant ones under As stress (Zvobgo et al., 2018). In the present study, the examined four PHT genes (*HvPHT1;1, HvPHT1;3, HvPHT1;4* and *HvPHT1;6*) were substantially upregulated under As stress in both genotypes as compared to control. However, mRNA transcripts of these PHT genes were relatively lower in Pu-9 than LJZ under As stress, which could account for less As uptake in Pu-9. The application of CaO NPs reduced the expression level of all PHT genes in As-exposed barley seedlings (Figure 7). Recently Zeshan et al. (2021) found that astaxanthin NPs could reduce the expression of Cd transporter genes in wheat plants under Cd stress conditions. In short, the current results showed that CaO NPs could alleviate barley As toxicity by reducing As uptake and transportation and improving antioxidant capacity.

**Figure 8:**
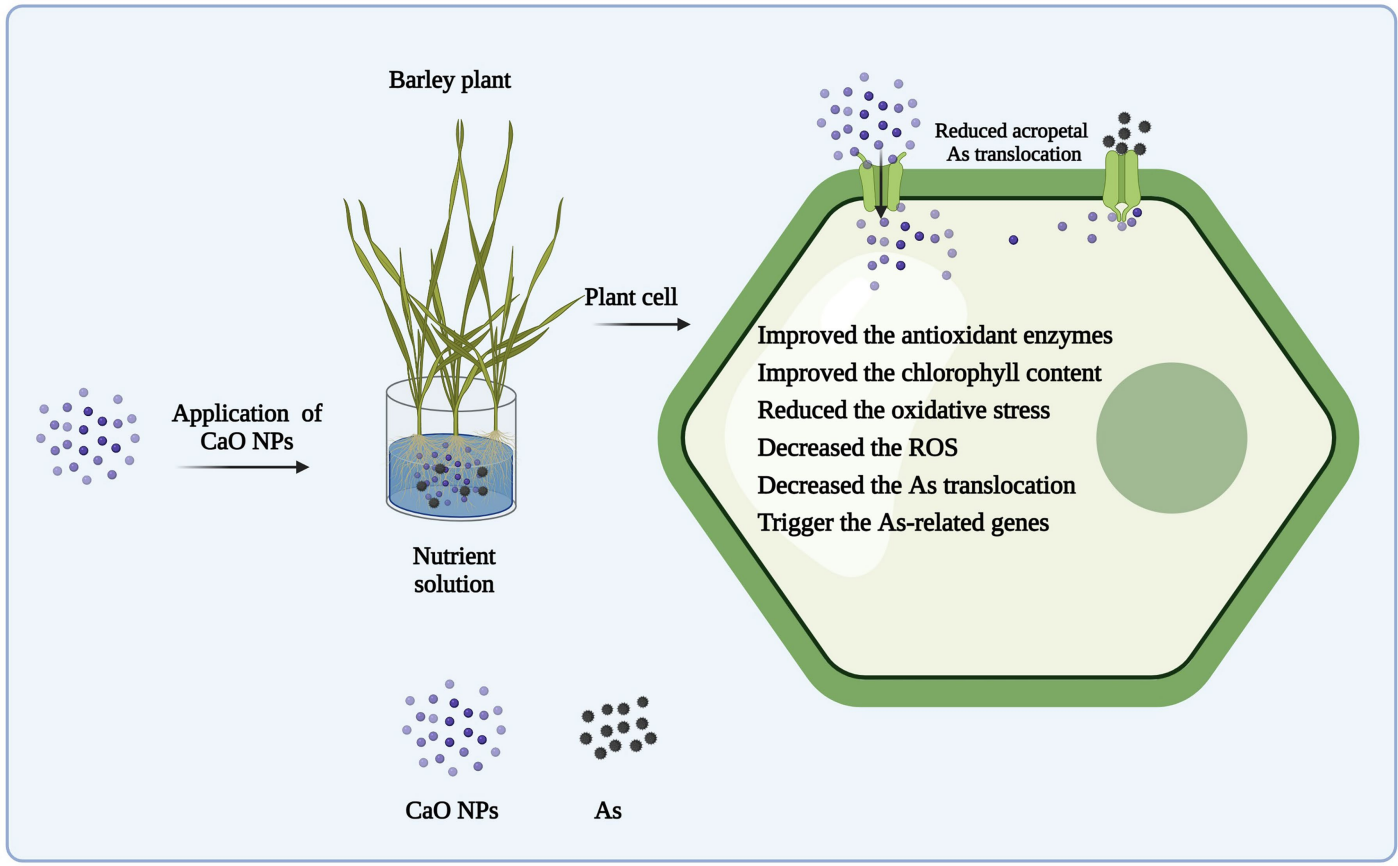
Working model showing how CaO NPs supplementation reduce As-induced phytotoxicities in barley seedlings. Initially, CaO NPs, after entering plant body, activate enzymatic antioxidants (i.e., SOD, POD, and CAT), which in turn help barley seedlings to manage metal-induced oxidative stress. Further, CaO NPs application supports plant growth by improving Ca^2+^ ions homeostasis and photosynthesis. In addition, CaO NPs reduce uptake, mobilization, and acropetal translocation of As by decreasing the transcript abundance of As transporting genes (*HvPHTs*), which ultimately helps plant to reprogram its normal physiological and developmental processes.

Taken together, our results show that CaO NPs alleviate As toxicity in barley by modulating different physiological and genetic pathways. More precisely, application of CaO NPs activates basal defense response by triggering enzymatic antioxidants, which subsequently reduce *in planta* ROS accumulation and rescue plants from As-induced oxidative stress. Further, CaO NPs application improves the photosynthetic efficacy by maintaining Ca^2+^ ions homeostasis. More interestingly, supplementation of CaO NPs downregulates expression levels of As transporters genes, and as a result ultimately reduce As mobilization and translocation within plant systems and protect barley seedlings from As-induced phytotoxicities. Conclusively, CaO NPs could protect barley seedlings from As stress by alleviating As-induced negative physiological alterations within plant systems (Figure 8).

## Conclusions

This study investigated the roles of CaO NPs in alleviating As toxicity in two barley genotypes differing in As tolerance. The roles of CaO NPs in alleviating As toxicity could be attributed to its enhancement of Ca uptake and ROS scavenging ability and reduction of As uptake and transportation from roots to shoots. Moreover, CaO NPS restricted the uptake and translocation of As through downregulating expressions of As transporter genes (*HvPHT1;1, HvPHT1;3, HvPHT1;4* and *HvPHT1;6*) in roots of barley seedlings. In addition, our results showed that the effect of CaO NPs in alleviating As toxicity or growth inhibition is genotype-dependent, with LJZ being more affected than Pu-9. This study provided further evidence that the application of CaO NPs is a potential solution for As-contaminated soils. However, more studies are required to decipher the mechanisms underlying the roles of CaO NPs in alleviating As toxicity in plants.

## Data Availability Statement

The original contributions presented in the study are included in the article/Supplementary Material, further inquiries can be directed to the corresponding author.

## Author Contributions

MN: conceptualization, methodology, writing—original draft, validation, and writing—review and editing. QL: data collection, formal analysis, validation, and writing—review and editing. MN and ZU: formal analysis, validation, and writing—review and editing. SA and TA: validation and writing—review and editing. FZ: co-supervision, methodology, and investigation. GZ: conceptualization, supervision, project administration, writing—review and editing, and funding acquisition. All authors contributed to the article and approved the submitted version.

## Funding

This work was supported by China Agriculture Research System (CARS-05) and Jiangsu Collaborative Innovation Center for Modern Crop Production (JCIC-MCP).

## Conflict of Interest

The authors declare that the research was conducted in the absence of any commercial or financial relationships that could be construed as a potential conflict of interest.

## Publisher’s Note

All claims expressed in this article are solely those of the authors and do not necessarily represent those of their affiliated organizations, or those of the publisher, the editors and the reviewers. Any product that may be evaluated in this article, or claim that may be made by its manufacturer, is not guaranteed or endorsed by the publisher.

## Footnotes

^1^ www.ncbi.nlm.nih.gov
