# Supplementary material for: Calcium Oxide Nanoparticles Have the Role of Alleviating Arsenic Toxicity of Barley
Source: Front Plant Sci. 2022 Mar 11;13:843795. doi: 10.3389/fpls.2022.843795 (PMC8963479; doi:10.3389/fpls.2022.843795)
Supplement: Supplementary file 1 [file Table_1.DOCX]

**Supplement table**

**Table S1** Primer sequences used for RT-PCR.

| Gene name | Forward sequence5’-3’ | Revers sequence5’-3’ |
| --- | --- | --- |
| *HvPHT1; 1* | ATCCGCCGAACCTGCATTAT | TTGAGCTGTTCAGTCGCCAT |
| *HvPHT1; 3* | GAAGTCGCTGGAGGAGATGT | GCAGCTGCAATAGCTAGCAC |
| *HvPHT1; 4* | ATGTTCACCTTCCTGGTGCC | ATAGCTAGCACGCACGAGTT |
| *HvPHT1; 6* | TGCGGATCAACCAAATCCCA | CTGTCACGATCAAGGAGGCA |
| *HvActin* | CGACAATGGAACCGGAATG | CCCTTGGCGCATCATCTC |
